# Supplementary material for: Optimized conditions for GTP loading of Ras
Source: J Biol Chem. 2025 Nov 7;301(12):110923. doi: 10.1016/j.jbc.2025.110923 (PMC12719659; doi:10.1016/j.jbc.2025.110923)
Supplement: Supporting Tables [file mmc1.pdf]

Supplemental Information for:

Optimized conditions for GTP loading of Ras

Kimberly J. Vish[1], Maxum E. Paul[1], Asha P. Rollins[2], and Titus J. Boggon[1,2,3*]

From: Departments of [1]Molecular Biophysics and Biochemistry and [2]Pharmacology, and the [3]Yale Cancer Center, Yale University, New Haven, CT, USA

* To whom correspondence should be addressed: titus.boggon@yale.edu

Supplemental Table 1. Conditions used for all loading replicates. Depending on the condition being tested some replicates were used in multiple figures as indicated by the figure legends.

Trial #	Mg^{2+} Addition	Amount loaded	Excess GTP	Time and temperature of loading	Storage Buffer	Days from loading	GTP	GDP	GMP	% GTP loaded	Change from Day 0
1	-	0.025 mM	10x	10 min 37˚C	20 mM Tris pH 8, 150 mM NaCl, 10 mM EDTA	0	4.1	3.4		54.7	0
2	-	0.025 mM	10x	10 min 37˚C	20 mM Tris pH 8, 150 mM NaCl, 10 mM EDTA	0	4.3	2.7		61.5	0
3	-	0.025 mM	10x	10 min 37˚C	20 mM Tris pH 8, 150 mM NaCl, 10 mM EDTA	0	3.0	2.5		54.6	0
4	-	0.025 mM	10x	10 min 37˚C	20 mM Tris pH 8, 150 mM NaCl, 10 mM EDTA	0	5.3	3.5		59.8	0
5	-	0.025 mM	10x	30 min 37˚C	20 mM Tris pH 8, 150 mM NaCl, 10 mM EDTA	0	6.2	3.3		65.5	0
6	-	0.025 mM	10x	30 min 37˚C	20 mM Tris pH 8, 150 mM NaCl, 10 mM EDTA	0	5.2	2.6		66.6	0
7	-	0.025 mM	10x	30 min 37˚C	20 mM Tris pH 8, 150 mM NaCl, 10 mM EDTA	0	5.6	3.2		64.2	0
8	-	0.025 mM	10x	30 min 37˚C	20 mM Tris pH 8, 150 mM NaCl, 10 mM EDTA	0	4.9	2.6		65.7	0
9	-	0.025 mM	10x	1 hr 37˚C	20 mM Tris pH 8, 150 mM NaCl, 10 mM EDTA	0	5.2	3.0		63.3	0
10	-	0.025 mM	10x	1 hr 37˚C	20 mM Tris pH 8, 150 mM NaCl, 10 mM EDTA	0	5.6	3.1		64.6	0
11	-	0.025 mM	10x	1 hr 37˚C	20 mM Tris pH 8, 150 mM NaCl, 10 mM EDTA	0	5.3	2.9		64.2	0
12	-	0.025 mM	10x	1 hr 37˚C	20 mM Tris pH 8, 150 mM NaCl, 10 mM EDTA	0	4.9	3.5		58.2	0
13	-	0.025 mM	10x	1 hr RT	20 mM Tris pH 8, 150 mM NaCl, 10 mM EDTA	0	4.9	2.4		67.5	0
14	-	0.025 mM	10x	1 hr RT	20 mM Tris pH 8, 150 mM NaCl, 10 mM EDTA	0	4.6	1.8		72.3	0
15	-	0.025 mM	10x	1 hr RT	20 mM Tris pH 8, 150 mM NaCl, 10 mM EDTA	0	5.0	2.7		65.2	0
16	-	0.025 mM	10x	1 hr RT	20 mM Tris pH 8, 150 mM NaCl, 10 mM EDTA	0	4.4	3.1		59.2	0

17	-	0.025 mM	10x	1 hr 0˚C	20 mM Tris pH 8, 150 mM NaCl, 10 mM EDTA	0	5.2	3.0		63.2	0
18	-	0.025 mM	10x	1 hr 0˚C	20 mM Tris pH 8, 150 mM NaCl, 10 mM EDTA	0	5.3	3.0		64.2	0
19	-	0.025 mM	10x	1 hr 0˚C	20 mM Tris pH 8, 150 mM NaCl, 10 mM EDTA	0	5.5	3.0		64.6	0
20	-	0.025 mM	10x	1 hr 0˚C	20 mM Tris pH 8, 150 mM NaCl, 10 mM EDTA	0	4.1	2.9		58.2	0
21	-	0.26 mM	10x	1 hr RT	20 mM Tris pH 8, 150 mM NaCl, 10 mM EDTA	0	13.7	4.0	2.4	68.2	0
22	-	0.26 mM	10x	1 hr RT	20 mM Tris pH 8, 150 mM NaCl, 10 mM EDTA	0	11.4	5.0	2.4	60.6	0
23	-	0.26 mM	10x	1 hr RT	20 mM Tris pH 8, 150 mM NaCl, 10 mM EDTA	0	13.4	3.6		78.8	0
24	-	0.26 mM	10x	1 hr RT	20 mM Tris pH 8, 150 mM NaCl, 10 mM EDTA	0	9.2	5.3	0.07	63.1	0
25	-	0.025 mM	10x	1 hr RT	20 mM Tris pH 8, 150 mM NaCl, 10 mM EDTA	0	11.2	9.1		55.2	0
26	+	0.025 mM	10x	1 hr RT	20 mM Tris pH 8, 150 mM NaCl, 10 mM EDTA	0	5.2	1.0		83.3	0
27	+	0.025 mM	10x	1 hr RT	20 mM Tris pH 8, 150 mM NaCl, 10 mM EDTA	0	5.6	1.2		82.6	0
28	+	0.025 mM	10x	1 hr RT	20 mM Tris pH 8, 150 mM NaCl, 10 mM EDTA	0	5.4	1.6		77.4	0
29	+	0.025 mM	10x	1 hr RT	20 mM Tris pH 8, 150 mM NaCl, 10 mM EDTA	0	4.7	1.9		71.1	0
30	+	0.025 mM	10x	1 hr RT	20 mM Tris pH 8, 150 mM NaCl, 10 mM EDTA	0	11.3	6.8		62.3	0
31	-	0.025 mM	10x	1 hr RT	20 mM Tris pH 8, 150 mM NaCl, 10 mM EDTA	0	11.2	9.1		55.2	0
32	-	0.025 mM	100x	10 min 37˚C	20 mM Tris pH 8, 150 mM NaCl, 10 mM EDTA	0	11.3	2.0		85.0	0
33	-	0.025 mM	100x	10 min 37˚C	20 mM Tris pH 8, 150 mM NaCl, 10 mM EDTA	0	12.9	3.2		80.2	0
34	-	0.025 mM	100x	10 min 37˚C	20 mM Tris pH 8, 150 mM NaCl, 10 mM EDTA	0	8.3	2.1		79.7	0
35	-	0.025 mM	100x	10 min 37˚C	20 mM Tris pH 8, 150 mM NaCl, 10 mM EDTA	0	8.1	2.1		79.3	0
36	-	0.025 mM	2.4x	10 min 37˚C	20 mM Tris pH 8, 150 mM NaCl,	0	4.1	4.9		45.6	0

					10 mM EDTA						
37	-	0.025 mM	2.4x	10 min 37˚C	20 mM Tris pH 8, 150 mM NaCl, 10 mM EDTA	0	4.3	5.1		45.8	0
38	-	0.025 mM	2.4x	10 min 37˚C	20 mM Tris pH 8, 150 mM NaCl, 10 mM EDTA	0	3.7	5.8		38.9	0
39	-	0.025 mM	2.4x	10 min 37˚C	20 mM Tris pH 8, 150 mM NaCl, 10 mM EDTA	0	4.8	7.1		39.9	0
40	+	0.025 mM	100x	10 min 37˚C	20 mM Tris pH 8, 150 mM NaCl, 10 mM EDTA	0	3.4	1.3		73.2	0
41	+	0.025 mM	100x	10 min 37˚C	20 mM Tris pH 8, 150 mM NaCl, 10 mM EDTA	0	7.0	2.1		77.0	0
42	+	0.025 mM	100x	10 min 37˚C	20 mM Tris pH 8, 150 mM NaCl, 10 mM EDTA	0	5.9	2.9		67.8	0
43	+	0.025 mM	100x	10 min 37˚C	20 mM Tris pH 8, 150 mM NaCl, 10 mM EDTA	0	5.9	2.1		73.7	0
44	-	0.025 mM	10x	1 hr 0˚C	20 mM Tris pH 8, 150 mM NaCl, 10 mM $MgCl_2$	0	3.6	4.2		46.2	0
45	-	0.025 mM	10x	1 hr 0˚C	20 mM Tris pH 8, 150 mM NaCl, 10 mM $MgCl_2$	0	4.0	3.0		57.1	0
46	-	0.025 mM	10x	1 hr 0˚C	20 mM Tris pH 8, 150 mM NaCl, 10 mM $MgCl_2$	0	3.0	4.6		39.3	0
47	-	0.025 mM	10x	1 hr 0˚C	20 mM Tris pH 8, 150 mM NaCl, 10 mM $MgCl_2$	0	5.2	3.7		58.1	0
48	-	0.025 mM	10x	1 hr 0˚C	20 mM Tris pH 8, 150 mM NaCl	0	5.5	3.2		63.1	0
49	-	0.025 mM	10x	1 hr 0˚C	20 mM Tris pH 8, 150 mM NaCl	0	6.0	3.4		64.1	0
50	-	0.025 mM	10x	1 hr 0˚C	20 mM Tris pH 8, 150 mM NaCl	0	6.2	3.5		64.1	0
51	-	0.025 mM	10x	1 hr 0˚C	20 mM Tris pH 8, 150 mM NaCl	0	5.0	3.2		61.1	0
52	-	0.025 mM	10x	1 hr 0˚C	20 mM Tris pH 8, 150 mM NaCl, 10 mM EDTA, 10 mM $MgCl_2$	0	2.7	4.6		36.8	0
53	-	0.025 mM	10x	1 hr 0˚C	20 mM Tris pH 8, 150 mM NaCl, 10 mM EDTA, 10 mM $MgCl_2$	0	2.6	4.8		35.0	0
54	-	0.025 mM	10x	1 hr 0˚C	20 mM Tris pH 8, 150 mM NaCl, 10 mM EDTA, 10 mM $MgCl_2$	0	2.7	5.1		34.8	0
55	-	0.025 mM	10x	1 hr 0˚C	20 mM Tris pH 8, 150 mM NaCl, 10 mM EDTA, 10 mM $MgCl_2$	0	2.7	5.0		35.4	0
56	-	0.025 mM	10x	1 hr 0˚C	20 mM Tris pH 8, 150 mM NaCl	0	11.0	7.3		60.2	0.0

57	-	0.025 mM	10x	1 hr 0˚C	20 mM Tris pH 8, 150 mM NaCl	1	6.4	11.6		35.6	-24.5
58	-	0.025 mM	10x	1 hr 0˚C	20 mM Tris pH 8, 150 mM NaCl	3	0.1	2.0		6.5	-53.6
59	-	0.025 mM	10x	10 min 37˚C	20 mM Tris pH 8, 150 mM NaCl	1	6.3	3.3		65.2	-19.9
60	-	0.025 mM	10x	10 min 37˚C	20 mM Tris pH 8, 150 mM NaCl	3	1.8	5.1		26.1	-58.9
61	-	0.025 mM	10x	10 min 37˚C	20 mM Tris pH 8, 150 mM NaCl	1	11.5	3.9		74.4	-5.8
62	-	0.025 mM	10x	10 min 37˚C	20 mM Tris pH 8, 150 mM NaCl	3	7.4	5.2		58.6	-21.6
63	-	0.025 mM	10x	10 min 37˚C	20 mM Tris pH 8, 150 mM NaCl	0	3.4	3.3		50.8	0.0
64	-	0.025 mM	10x	10 min 37˚C	20 mM Tris pH 8, 150 mM NaCl	1	2.7	4.2		39.4	-11.4
65	-	0.025 mM	10x	10 min 37˚C	20 mM Tris pH 8, 150 mM NaCl	3	1.8	5.1		26.1	-24.7
66	-	0.025 mM	10x	10 min 37˚C	20 mM Tris pH 8, 150 mM NaCl, 10 mM EDTA	1	4.4	3.6		55.0	0.3
67	-	0.025 mM	10x	10 min 37˚C	20 mM Tris pH 8, 150 mM NaCl, 10 mM EDTA	3	3.8	3.5		52.1	-2.7
68	-	0.025 mM	10x	10 min 37˚C	20 mM Tris pH 8, 150 mM NaCl, 10 mM EDTA	1	3.0	2.4		55.9	-5.5
69	-	0.025 mM	10x	10 min 37˚C	20 mM Tris pH 8, 150 mM NaCl, 10 mM EDTA	3	2.5	2.2		53.8	-7.7
70	-	0.025 mM	10x	10 min 37˚C	20 mM Tris pH 8, 150 mM NaCl, 10 mM EDTA	1	2.9	2.4		54.3	-0.3
71	-	0.025 mM	10x	10 min 37˚C	20 mM Tris pH 8, 150 mM NaCl, 10 mM EDTA	3	2.3	2.1		52.3	-2.3
72	-	0.025 mM	10x	10 min 37˚C	20 mM Tris pH 8, 150 mM NaCl, 10 mM EDTA	0	4.6	2.9		61.0	0.0
73	-	0.025 mM	10x	10 min 37˚C	20 mM Tris pH 8, 150 mM NaCl, 10 mM EDTA	1	4.0	3.2		55.1	-5.9
74	-	0.025 mM	10x	10 min 37˚C	20 mM Tris pH 8, 150 mM NaCl, 10 mM EDTA	3	3.3	3.2		50.6	-10.4
75	-	0.025 mM	10x	1 hr 0˚C	20 mM Tris pH 8, 150 mM NaCl, 10 mM $MgCl_2$	1	1.9	4.7		28.3	-17.8
76	-	0.025 mM	10x	1 hr 0˚C	20 mM Tris pH 8, 150 mM NaCl, 10 mM $MgCl_2$	3	0.8	4.9		13.6	-32.6
77	-	0.025 mM	10x	10 min 37˚C	20 mM Tris pH 8, 150 mM NaCl, 10 mM $MgCl_2$	0	3.2	4.0		44.7	0.0
78	-	0.025 mM	10x	10 min 37˚C	20 mM Tris pH 8, 150 mM NaCl, 10 mM $MgCl_2$	1	1.8	5.1		25.5	-19.2

79	-	0.025 mM	10x	10 min 37˚C	20 mM Tris pH 8, 150 mM NaCl, 10 mM $MgCl_2$	3	0.9	5.4		14.6	-30.1
80	-	0.025 mM	10x	10 min 37˚C	20 mM Tris pH 8, 150 mM NaCl, 10 mM $MgCl_2$	0	4.6	3.1		59.5	0.0
81	-	0.025 mM	10x	10 min 37˚C	20 mM Tris pH 8, 150 mM NaCl, 10 mM $MgCl_2$	1	2.5	4.4		36.4	-23.0
82	-	0.025 mM	10x	10 min 37˚C	20 mM Tris pH 8, 150 mM NaCl, 10 mM $MgCl_2$	3	1.3	3.9		24.5	-34.9
83	-	0.025 mM	10x	10 min 37˚C	20 mM Tris pH 8, 150 mM NaCl, 10 mM $MgCl_2$	0	3.9	2.7		59.1	0.0
84	-	0.025 mM	10x	10 min 37˚C	20 mM Tris pH 8, 150 mM NaCl, 10 mM $MgCl_2$	1	2.3	5.0		31.4	-27.7
85	-	0.025 mM	10x	10 min 37˚C	20 mM Tris pH 8, 150 mM NaCl, 10 mM $MgCl_2$	3	1.5	5.1		22.9	-36.3
86	-	0.025 mM	10x	1 hr 0˚C	20 mM Tris pH 8, 150 mM NaCl, 10 mM EDTA, 10 mM $MgCl_2$	0	3.0	3.6		45.2	0.0
87	-	0.025 mM	10x	1 hr 0˚C	20 mM Tris pH 8, 150 mM NaCl, 10 mM EDTA, 10 mM $MgCl_2$	1	2.9	4.2		40.7	-4.5
88	-	0.025 mM	10x	1 hr 0˚C	20 mM Tris pH 8, 150 mM NaCl, 10 mM EDTA, 10 mM $MgCl_2$	3	1.0	5.2		16.2	-28.9
89	-	0.025 mM	10x	10 min 37˚C	20 mM Tris pH 8, 150 mM NaCl, 10 mM EDTA, 10 mM $MgCl_2$	0	3.1	3.8		45.1	0.0
90	-	0.025 mM	10x	10 min 37˚C	20 mM Tris pH 8, 150 mM NaCl, 10 mM EDTA, 10 mM $MgCl_2$	1	2.1	4.8		30.3	-14.8
91	-	0.025 mM	10x	10 min 37˚C	20 mM Tris pH 8, 150 mM NaCl, 10 mM EDTA, 10 mM $MgCl_2$	3	0.9	5.4		14.7	-30.3
92	-	0.025 mM	10x	10 min 37˚C	20 mM Tris pH 8, 150 mM NaCl, 10 mM EDTA, 10 mM $MgCl_2$	0	2.7	3.5		43.1	0.0
93	-	0.025 mM	10x	10 min 37˚C	20 mM Tris pH 8, 150 mM NaCl, 10 mM EDTA, 10 mM $MgCl_2$	1	2.0	4.4		31.0	-12.1
94	-	0.025 mM	10x	10 min 37˚C	20 mM Tris pH 8, 150 mM NaCl, 10 mM EDTA, 10 mM $MgCl_2$	3	0.9	5.3		14.6	-28.5
95	-	0.025 mM	10x	10 min 37˚C	20 mM Tris pH 8, 150 mM NaCl, 10 mM EDTA, 10 mM $MgCl_2$	0	3.7	4.4		45.7	0.0
96	-	0.025 mM	10x	10 min 37˚C	20 mM Tris pH 8, 150 mM NaCl, 10 mM EDTA, 10 mM $MgCl_2$	1	2.4	4.7		33.6	-12.2
97	-	0.025 mM	10x	10 min 37˚C	20 mM Tris pH 8, 150 mM NaCl, 10 mM EDTA, 10 mM $MgCl_2$	3	1.2	4.6		20.7	-25.1

Supplemental Table 2. The conditions and replicates of the nucleotide content of the loaded K-Ras and N-Ras isoforms.

Loaded Ras Isoforms										
**Trial #**	**Mg2+ Addition**	**Amount Loaded**	**Excess GTP**	**Time and temperature of loading**	**Storage Buffer**	**Isoform**	**GTP**	**GDP**	**GMP**	**% GTP loaded**
98	-	0.025 mM	10x	10 min 37˚C	20 mM Tris pH 8, 150 mM NaCl, 10 mM EDTA	K-Ras	**3.8**	**2.8**		**57.1**
99	-	0.025 mM	10x	10 min 37˚C	20 mM Tris pH 8, 150 mM NaCl, 10 mM EDTA	K-Ras	4.1	3.9		51.3
100	-	0.025 mM	10x	10 min 37˚C	20 mM Tris pH 8, 150 mM NaCl, 10 mM EDTA	K-Ras	3.4	3.3		51.3
101	-	0.025 mM	10x	10 min 37˚C	20 mM Tris pH 8, 150 mM NaCl, 10 mM EDTA	K-Ras	3.2	2.8		53.4
102	-	0.025 mM	10x	10 min 37˚C	20 mM Tris pH 8, 150 mM NaCl, 10 mM EDTA	N-Ras	2.2	1.4		61.1
103	-	0.025 mM	10x	10 min 37˚C	20 mM Tris pH 8, 150 mM NaCl, 10 mM EDTA	N-Ras	3.3	2.4		57.7
104	-	0.025 mM	10x	10 min 37˚C	20 mM Tris pH 8, 150 mM NaCl, 10 mM EDTA	N-Ras	3.1	2.1		59.5
105	-	0.025 mM	10x	10 min 37˚C	20 mM Tris pH 8, 150 mM NaCl, 10 mM EDTA	N-Ras	3.3	2.2		60.2

Supplemental Table 3. Nucleotide content of purified wild-type and mutant H-Ras.

Purified Cancer Mutations				
**Mutation**	**GTP**	**GDP**	**GMP**	**% GTP**
WT	1.7	16.9	9.8	6.0
WT	0.8	9.8	7.9	4.3
WT	2.5	37.5	13.8	4.6
WT	2.3	38.0	14.3	4.3
G12V	15.0	13.4	14.0	35.4
G12V	11.8	21.6	17.9	23.1
G12V	10.7	24.7	10.3	23.4
G12V	14.1	29.8	16.1	23.6
G13R	2.5	11.5	10.6	10.1
G13R	8.5	34.8	17.5	14.0
G13R	6.5	38.3	19.4	10.1
G13R	7.4	29.9	15.1	14.2
Q61L	21.2	20.2	26.7	31.1
Q61L	18.4	19.4	24.6	29.4
Q61L	18.1	23.7	10.2	34.9
Q61L	19.3	23.7	19.3	31.0

Supplemental Table 4. The conditions and replicates of the nucleotide content of loaded H-Ras-G12V.

Loaded Cancer Mutations										
Trial #	Mg2+ Addition	Amount Loaded	Excess GTP	Time and temperature of loading	Storage Buffer	Mutation	GTP	GDP	GMP	% GTP loaded
106	-	0.025 mM	10x	10 min 37˚C	20 mM Tris pH 8, 150 mM NaCl, 10 mM EDTA	G12V	14.3	25.7		35.8
107	-	0.025 mM	10x	10 min 37˚C	20 mM Tris pH 8, 150 mM NaCl, 10 mM EDTA	G12V	8.6	9.5		47.5
108	-	0.025 mM	10x	10 min 37˚C	20 mM Tris pH 8, 150 mM NaCl, 10 mM EDTA	G12V	6.2	11.6		34.8
109	-	0.025 mM	10x	10 min 37˚C	20 mM Tris pH 8, 150 mM NaCl, 10 mM EDTA	G12V	3.6	10.4		25.4
